# *Actívatexto:* Feasibility and Acceptability of a Mobile Intervention That Promotes Smoking Cessation and Physical Activity among Latinos

**DOI:** 10.1158/2767-9764.CRC-23-0519

**Published:** 2024-04-08

**Authors:** Daimarelys Lara, Edgar I. Alaniz-Cantú, Simran Siddalingaiaha, Igor Oliveira, Arlette Chávez-Iñiguez, Elisa DeJesus, Daniel Fuller, David X. Marquez, Elizabeth Vásquez, Dongmei Li, Scott McIntosh, Deborah J. Ossip, Ana Paula Cupertino, Francisco Cartujano-Barrera

**Affiliations:** 1 Department of Public Health Sciences, University of Rochester Medical Center, Rochester, New York.; 2 Language Services, Ibero American Action League, Rochester, New York.; 3 Department of Community Health and Epidemiology, University of Saskatchewan, Saskatoon, Canada.; 4 Department of Kinesiology and Nutrition, University of Illinois Chicago, Chicago, Illinois.; 5 Department of Epidemiology and Biostatistics, SUNY University at Albany, Albany, New York.; 6 Clinical and Translational Science Institute, University of Rochester Medical Center, Rochester, New York.; 7 Department of Surgery, University of Rochester Medical Center, Rochester, New York.

## Abstract

The purpose of this study was to assess the feasibility and acceptability of a mobile intervention that promotes smoking cessation and physical activity among Latinos living in the United States. Twenty Latino adults who smoked and did not meet recommended levels of physical activity were recruited using community-based recruitment strategies. Participants received *Actívatexto*, a theory-based, culturally accommodated, 12-week text messaging intervention (available in English and Spanish) that promotes smoking cessation and physical activity. *Actívatexto* integrates four components: (i) a text messaging program, (ii) a wearable device, (iii) an online dashboard where the research team manages participants’ incoming and outgoing data from both the text messaging program and wearable device, and (iv) nicotine replacement therapies (NRT). We assessed self-reported 7-day point prevalence smoking abstinence at month 3, weekly minutes of moderate to vigorous physical activity (MVPA), pharmacotherapy adherence, and satisfaction. At baseline, participants’ mean age was 47.3 years old (SD 16.0), 55% of participants were female, and 60% indicated that Spanish was their language of preference. Seventy-five percent of participants smoked 1–10 cigarettes daily and 35% smoked their first cigarette within 5 minutes after waking up. At month 3, 14 participants (70%) self-reported 7-day point prevalence smoking abstinence (90% follow-up rate). Fitbit-assessed mean minutes of MVPA per week increased from 113 (SD 127; range: 0–399) at week 1 to 177 (SD 163; range: 0–513) at month 3 (*P* = 0.15). Sixteen participants (88.9%, 16/18) reported NRT use in the past 90 days. Most participants reported being extremely satisfied or satisfied with the intervention (88.9%, 16/18).

**Significance:**

*Actívatexto* resulted in a noteworthy cessation rate at month 3 (70%), increased mean weekly minutes of MVPA, produced high use of NRT, and was well received by participants. Additional testing in a randomized clinical trial is warranted to assess the efficacy of the intervention.

## Introduction

Latinos are the largest minority group in the United States, accounting for 17.4% of the U.S. population, and are projected to grow to nearly 30% by 2060 (1, 2). Of the approximately 55 million Latinos who live in the Union States, over 4 million (8.0%) smoke cigarettes (3). Smoking among Latino adults is not homogeneous, with strikingly high smoking rates among Puerto Ricans (women 32.6%, men 35.0%) and Cubans (women 21.9%, men 31.3%; ref. 4). Overall, Latino adults experience multiple tobacco-related disparities. Compared with Black and White adults, Latino adults receive less advice to quit smoking and have less access to cessation resources (e.g., behavioral counseling and pharmacotherapy; refs. 5–10). Specific barriers when accessing cessation resources include a paucity of resources in Spanish, lack of cultural sensitivity, mistrust of the health care system, and limited knowledge regarding cessation resources (10). Although barriers to accessing treatment exist, Latino adults are interested in using behavioral counseling and pharmacotherapy to support quit attempts (11–16).

Research on developing and implementing smoking cessation interventions among Latino adults has been conducted (15–18). For instance, Cartujano-Barrera and colleagues pilot tested a culturally tailored smoking cessation text messaging intervention among Latino adults who smoke (16). The text messaging intervention offered a promising strategy to increase the use of nicotine replacement therapies (NRT), produced high satisfaction, and resulted in a notable cessation rate (30%) among Latino adults (16). To the best of our knowledge, no study has leveraged the role of physical activity in enhancing cessation rates among Latino adults. Moderate to vigorous physical activity (MVPA) is associated with outcomes that predict smoking cessation, including management of relapse/lapse, acute relief from nicotine withdrawal, and greater self-efficacy to quit smoking (19–22). MVPA also lessens fear about weight gain, a frequent concern among individuals who smoke (23). In 2019, Ussher and colleagues conducted a Cochrane review on exercise and physical activity interventions for smoking cessation, concluding that there is insufficient evidence on the efficacy of incorporating exercise and physical activity into smoking cessation treatment (24). Estimates of treatment effect were of low or very low certainty because of concerns about risk of bias and imprecision (24). Nevertheless, four studies have shown higher cessation rates among individuals who smoke assigned to an intervention that promotes MVPA compared with control (25–28). However, these trials were hampered by limitations including small sample sizes, absence of men, exclusion of non-English speaking individuals, or no representation of racial and ethnic minorities (25–28). Moreover, these studies have relied on highly structured, supervised, vigorous exercise at research-based fitness facilities, which may not be broadly disseminated. Furthermore, some of these studies used self-reported data on physical activity. The purpose of this pilot study was to assess the feasibility and acceptability of *Actívatexto*, a mobile intervention that promotes smoking cessation and physical activity among Latino adults living in the United States.

## Materials and Methods

### Study Design

This was a single-arm pilot study with 20 Latino adults who smoke and do not meet recommended levels of physical activity. Participants received *Actívatexto*, a culturally accommodated mobile intervention that promotes smoking cessation and physical activity. Study procedures were approved and monitored by the University of Rochester Medical Center (URMC) Institutional Review Board (protocol number STUDY00007515). The study was conducted in accordance with the Declaration of Helsinki. Participants were compensated with a $30 gift card for enrolling in the study and a $50 gift card for completing the 12-week follow-up assessment.

### Recruitment

Recruitment was conducted in New York State by a team of bilingual (English and Spanish) trained research staff between January and May 2023. Recruitment strategies included study presentations in community-based organizations, malls, Latino festivals, referrals from the Wilmot Tobacco Cessation Center at the URMC, and word of mouth from community partners.

### Eligibility

Individuals were eligible if they (i) self-identified as Latino(a) and/or Hispanic; (ii) knew how to speak and read English and/or Spanish; (iii) were 21 years of age or older; (iv) did not meet the recommended 150 minutes of MVPA per week (measured by the International Physical Activity Questionnaire; refs. 29, 30); (v) smoked at least 3 days per week; (vi) were interested in quitting smoking in the next 30 days; (vii) had a cellphone with text messaging capability; (viii) knew how to utilize text messages; (ix) were willing to wear a wearable device to monitor physical activity; and (x) were willing to complete all study visits. Individuals were not eligible if they (i) used tobacco products other than cigarettes in the past 30 days (including e-cigarettes); (ii) were pregnant or breastfeeding; (iii) were planning to move from their current residence in the upcoming 6 months; (iv) had another household member enrolled in the study; and (v) were unable to become more physically active or engage in a fitness appraisal [as determined by the Physical Activity Readiness Questionnaire, PAR-Q (31, 32)]. The PAR-Q is a simple questionnaire designed to determine whether or not it is safe for an individual to engage in physical activity (31, 32).

### Screening and Consent

Trained research staff conducted the eligibility assessment over the phone. Individuals who were eligible to participate in the study were scheduled for a baseline appointment in-person or by telephone. During the baseline appointment, staff discussed all aspects of study participation, answered any questions, and guided individuals through the process of written informed consent. Eligibility assessment and consent were available in the participant's language of preference, either English or Spanish.

### Intervention

*Actívatexto* is a theory-based, culturally accommodated, 12-week text messaging intervention (available in English and Spanish) that promotes both smoking cessation and physical activity. *Actívatexto* integrates four components: (i) a text messaging program that promotes both smoking cessation and physical activity, (ii) wearable devices to monitor physical activity, (iii) an online dashboard where the research team manages participants’ incoming and outgoing data from both the text messaging program and wearable devices, and (iv) smoking cessation pharmacotherapy (i.e., NRTs). *Actívatexto* builds upon *Decídetexto*, a mobile intervention that solely promotes smoking cessation among Latinos (17, 18). *Actívatexto* was informed by (i) social cognitive theory, (ii) literature reviews, (iii) feedback from key stakeholders, tobacco treatment specialists, and exercise physiologists, and (iv) interviews with Latino adults who smoke (33). The intervention was refined by a multidisciplinary Community Advisory Board (CAB), where each member represented different Latin American countries to ensure the cultural and linguistic congruency of each intervention component.

#### Text Messages

Similar to *Decídetexto* (17, 18), the text messaging component of *Actívatexto* allows three levels of interactivity: (i) prescheduled standard messages, (ii) keyword-triggered standard messages, and (iii) counselor-personalized responses.

##### Prescheduled Standard Messages

The text messaging library consisted of messages covering 10 themes: education, intratreatment social support, extra treatment social support, coping with triggers, stimulus control, vicarious experience, social norms, relapse prevention, reward, and logistics. The text messages were delivered according to an algorithm based on four sequential phases of the quitting process: (i) Pre-quit (30 days), (ii) Quit-Day, (iii) Post-quit Intensive (28 days), and (iv) Post-quit Maintenance (6 weeks). *Actívatexto* also included a Relapse track (8 days).

##### Keyword-triggered Standard Messages

These messages consisted of automated immediate responses sent to participants who texted one of the following keywords: (i) Family, (ii) Stress, (iii) Crave, (iv) Patch, and (v) Gum. In addition, throughout the 12-week program, participants received six response-triggered (YES or NO) messages to assess their smoking status (e.g., Have you smoked a cigarette (even a puff) in the last 7 days? Text YES or NO). If participants indicated that they were smoking, the intervention guided them to selecting a new quit date. Participants could stop receiving the text messages at any moment by texting the keyword “Stop”.

##### Personalized Responses

The intervention encouraged participants to text any concerns and/or questions to the program (e.g., on day 1 of the text messaging intervention, participants received the following message: “Hi, I am your counselor. Feel free to text me anytime—I am here to help you Monday through Friday from 8 AM to 6 PM. When you text me, I will reply as soon as possible”). Research staff, who are also trained tobacco treatment specialists, answered these messages following standard protocols (e.g., answering questions on pharmacotherapy use and side effects). Staff monitored and triaged queries daily, responding within 48 hours of receipt of text messages.

#### Wearable Devices

Participants were provided with a Fitbit Versa 4 device. Fitbit devices provide acceptably accurate measures of MVPA (34–37). Providing participants with a Fitbit device homogenized the tracking of physical activity compared with providing other wearable devices (e.g., Apple Watch) or using smartphone applications (e.g., Health by iOS), which would not have been standardized across all operating systems (e.g., Google's Android and Apple's iOS). Research staff mailed the Fitbit devices to each participant and helped them set up their accounts. Participants were instructed to wear the Fitbit on their wrist daily and received weekly messages regarding their weekly levels of physical activity. The goal was for participants to complete at least 150 minutes of MVPA each week: 150 minutes of moderate activity or 75 minutes of vigorous activity, or a combination of both. Finally, Fitbit devices provide some motivational components for physical activity including reminders about long inactive periods and a celebration on the device when individuals step goal for the day has been reached.

#### Online Dashboard

*Actívatexto* relied on an online dashboard where the research staff managed participants’ incoming and outgoing data from both the text messaging program and wearable devices. *Actívatexto* synchronized the Fitbit Versa 4 devices with the text messaging platform. This synchronization allowed research staff to monitor participants’ physical activity on a weekly basis. Data were used to send automated individualized text messages to participants indicating their progression toward completing the 150 minutes of MVPA per week.

#### NRT

The use of NRT in this study followed the Clinical Practice Guideline for Tobacco Use and Dependence (38). Nicotine patches, gum, and lozenges were offered to participants at no cost. Participants who smoked more than 10 cigarettes per day (CPD) were offered ten weeks of nicotine patches (21 mg nicotine patches to be used during the first 6 weeks, followed by 14 mg nicotine patches for 2 weeks, and 7 mg patches for the last 2 weeks). Participants who smoked between six and 10 CPD were offered 8 weeks of nicotine patches (14 mg nicotine patches to be used during the first 6 weeks, followed by 7 mg patches for the last 2 weeks), gum (2 mg nicotine gum), or lozenges (2 mg nicotine lozenges). Participants who smoked five or less CPD were offered 6 weeks of nicotine patches (7 mg nicotine patches), gum (2 mg nicotine gum), or lozenges (2 mg nicotine lozenges). The gum was available in the following flavors: fruit chill, cinnamon, and mint. Participants who were interested in using NRT received them via postal mail. This approach has been used in previous studies (16–18).

### Assessments

Assessments were completed in the language of preference of the participant, either English or Spanish. The baseline survey collected sociodemographic variables such as age, gender, sexual orientation, education level, language of preference, and country of birth. Smoking-related variables collected at baseline included the number of CPD, time to first cigarette, use of menthol cigarettes, if they made a quit attempt in the previous year, smoking in the social network, and their self-efficacy for abstinence [measured by the smoking self-efficacy questionnaire (SEQ-12)] (39, 40). The SEQ-12 consists of 12 items, and each item is rated on a 5-point Likert scale (1 = “Not at all sure” to 5 = “Absolutely sure”; refs. 39, 40). SEQ-12 scores range from 12 to 60 with higher scores indicating greater self-efficacy (39, 40). Finally, participants were asked about their self-efficacy for physical activity [measured by the self-efficacy for physical activity (SEPA) scale] (41, 42). The SEPA scale consists of 5 items, and each item is rated on a 5-point Likert scale (1 = “Not confident” to 5 = “Extremely confident”; refs. 41, 42). SEPA scores range from 5 to 25 with higher scores indicating greater self-efficacy (41, 42).

The Fitbit Versa 4 devices provide data on “active minutes.” Active minutes, calculated within the proprietary algorithms of Fitbit, are defined as the time spent doing MVPA. For moderate-intensity physical activity, the heart rate should be between 64% and 76% of the maximum heart rate (43). For vigorous-intensity physical activity, the heart rate should be between 77% and 93% of the maximum heart rate (43). The maximum heart rate is a custom rate based on age and is the subtraction of age in years from 220 (43). Each minute of moderate-intensity physical activity equals one active minute. Each minute of vigorous-intensity physical activity equals two active minutes. These measures are based on guidelines for physical activity (44–46).

Adherence to Fitbit Versa 4 wear was defined as the total percentage of device wear during the intervention period (47). Consistent with previous criteria, Fitbit wear (i.e., whether or not a participant was considered to have worn the device for a particular day) was determined by an objective record of 100 steps in a day (48–50).

Three months after enrollment, a follow-up assessment was conducted. The primary outcome was self-reported 7-day point prevalence abstinence (not smoking any cigarettes in the past 7 days). Secondary outcomes were pharmacotherapy adherence, self-efficacy, and satisfaction. Pharmacotherapy use was assessed by the question “Have you used the nicotine patches/gum/lozenges in the last 3 months?” (Yes/No). If a participant indicated NRT use in the last 3 months, the following question was asked “In the last 3 months, how many days did you use the nicotine patches/gum/lozenges?” Self-efficacy for smoking cessation and physical activity were measured with the SEQ-12 and SEPA scale, respectively (39–42). Acceptability measures included questions such as “How satisfied are you with the smoking cessation text messaging program?” (1 = “Extremely unsatisfied” to 5 = “Extremely satisfied”), “Would you recommend this program to a friend?” (Yes/No), “In general, how much did this program help you quit smoking cigarettes?” (1 = “Not at all” to 4 = “A lot”), and “In general, how much did this program help you be physically active?” (1 = “Not at all” to 4 = “A lot”).

### Analysis

Frequencies were calculated for categorical variables. Means and SDs were calculated for continuous variables. Primary analysis on smoking cessation was conducted using the Russell standard, treating those lost to follow-up as participants who continued smoking (51). The secondary analyses on MVPA, pharmacotherapy use, self-efficacy, and satisfaction were conducted using complete case analysis, in which missing values in the outcome were considered to be missing. Weekly minutes of MVPA, self-efficacy for smoking cessation, and SEPA at baseline and follow-up were compared using paired sample *t* tests to examine differences.

### Data Availability Statement

The data generated in this study are available upon request from the corresponding author.

## Results

**FIGURE 1:**
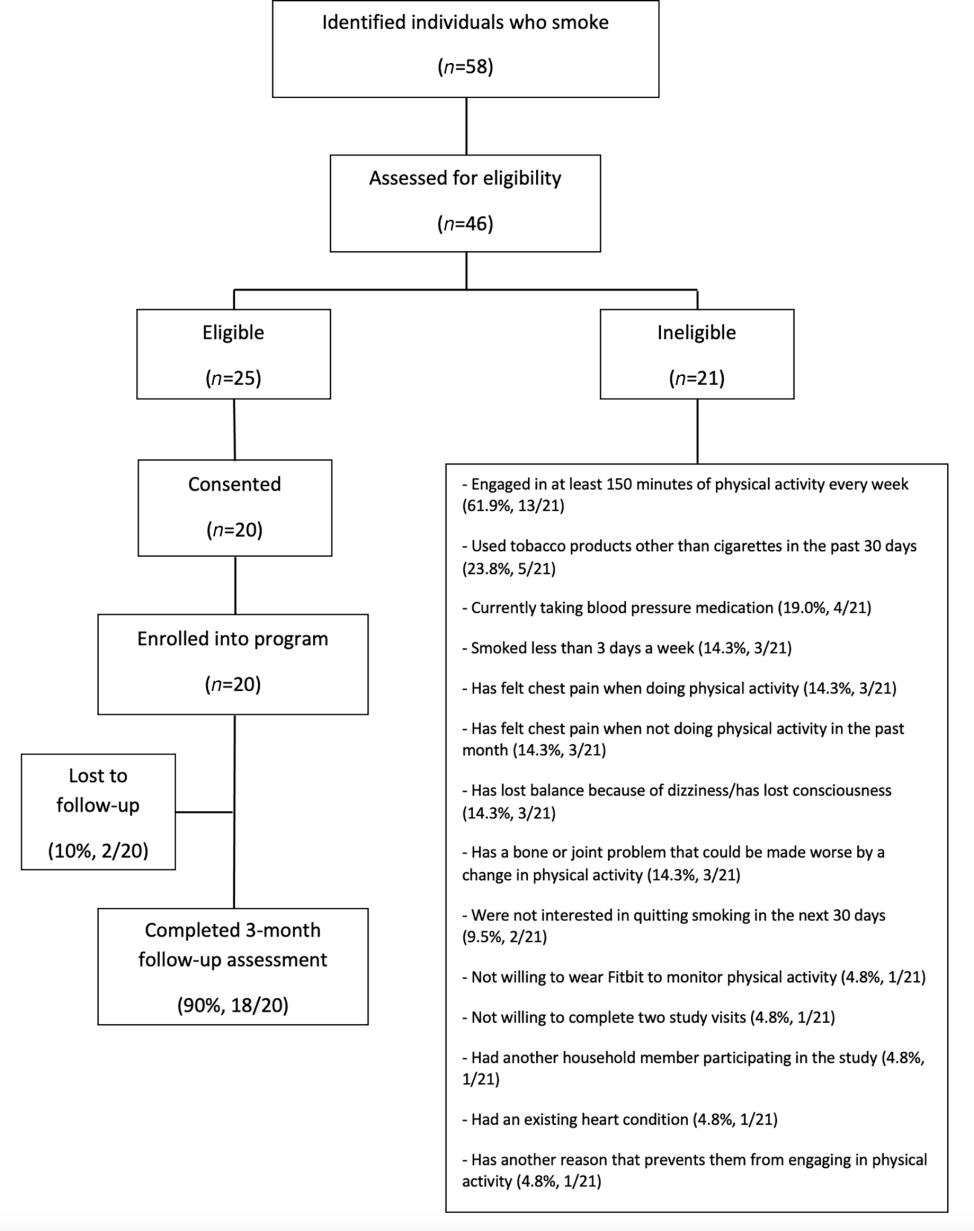
Study design flow diagram.

Fifty-eight Latino adults who smoked were identified. Among these, 46 were assessed for eligibility; 25 were eligible to participate in the study. Twenty Latino adults who smoked and did not meet the recommended levels of MVPA consented to participate in the study and completed the baseline assessment (Fig. 1).

At baseline, mean age among participants was 47.3 years old (SD 16.0), 55% of participants were female, 75% were heterosexual or straight, 80% had completed high school or lower educational level, and 60% indicated that Spanish was their language of preference. Eight participants (40%) were born in Cuba, 6 (30%) in the United States, 3 (15%) in Puerto Rico, and one each from three other countries. Most participants (75%) smoked 1–10 cigarettes daily, 35% smoked their first cigarette within 5 minutes after waking up, 60% used menthol cigarettes and, on average, 3 of their 5 closest friends smoke (mean 3.3, SD 1.7). Most participants (75%) tried to quit smoking in the past year and 60% have used pharmacotherapy and electronic cigarettes for smoking cessation in their lifetime. The mean scores of self-efficacy for smoking cessation and physical activity were 30.2 (SD 10.6) and 12.3 (SD 3.9), respectively (Table 1). Seventeen participants (85%) requested NRT at baseline: 9 (45%) requested patches, 7 (35%) requested gum, and 1 (5%) requested lozenges.

**TABLE 1:** Baseline characteristics of participants

Characteristics	*n* = 20 (%)
Age, mean (SD)	47.3 (16.0)
Gender	
Female	11 (55.0%)
Male	9 (45.0%)
Sexual orientation	
Heterosexual or straight	15 (75.0%)
Homosexual or gay	4 (20.0%)
Bisexual	1 (5.0%)
Education level	
High school or less	16 (80.0%)
More than high school	4 (20.0%)
Language of preference	
Spanish	12 (60.0%)
English	8 (40.0%)
Country of birth	
Cuba	8 (40.0%)
United States	6 (30.0%)
Puerto Rico	3 (15.0%)
Venezuela	1 (5.0%)
Panama	1 (5.0%)
Dominican Republic	1 (5.0%)
Smoking pattern	
Daily, 1–10 CPD	15 (75.0%)
Daily, 11–20 CPD	5 (25.0%)
Time to first cigarette	
≤5 minutes after waking up	7 (35.0%)
>5 minutes after waking up	13 (65.0%)
Use of menthol cigarettes	12 (60.0%)
Number of 5 closest friends who smoke, mean (SD)	3.3 (1.7)
Quit attempt in past year	15 (75.0%)
Use of cessation pharmacotherapy in the past^a^	12 (60.0%)
Use of E-cigarettes for cessation in the past	12 (60.0%)
Self-efficacy for abstinence^b^, mean (SD)	30.2 (10.6)
Self-efficacy for physical activity^c^, mean (SD)	12.3 (3.9)

Abbreviations: CPD: cigarettes per day; SD: standard deviation.

^a^Cessation pharmacotherapy included the nicotine patch, nicotine gum, nicotine lozenge, nicotine nasal spray, nicotine inhaler, Chantix/Varenicline, and Zyban/Bupropion.

^b^Self-efficacy for abstinence was measured by the smoking self-efficacy questionnaire (SEQ-12). SEQ-12 scores range from 12 to 60 with higher scores indicating greater self-efficacy.

^c^Self-efficacy for physical activity was measured by the SEPA scale. SEPA scores range from 5 to 25 with higher scores indicating greater self-efficacy.

Fitbit-assessed mean minutes of MVPA per week increased from 113 (SD 127; range: 0–399) at week 1 to 177 (SD 163; range: 0–513) at month 3, although the difference was not statistically significant (*P* = 0.15). On average, participants adhered to wearing their Fitbit Versa 4 device 69.2% of the days during the 3-month period.

At month 3, 14 participants (70%) self-reported 7-day point prevalence abstinence. The follow-up rate was 90% (18/20). Sixteen participants self-reported using NRT for at least one day. Using an analysis with participants who self-reported using NRT for at least one day, on average, participants self-reported using NRT for 39.6 days (SD 31.7, range: 3–90). Mean scores of self-efficacy for smoking cessation increased significantly from 30.2 (SD 10.6) at baseline to 47.8 (SD 13.0) at follow-up (*P* < 0.01). Mean scores of SEPA increased from 12.3 (SD 3.9) at baseline to 14.6 (SD 4.4) at follow-up, although the difference was not statistically significant (*P* = 0.2). Most participants reported being extremely satisfied or satisfied with the intervention (88.9%, 16/18), and said they would recommend the program to a friend (94.4%, 17/18). Moreover, most participants reported that the program helped them a lot to quit smoking (88.9%, 16/18) and be physically active (83.3%, 15/18).

## Discussion

To the best of our knowledge, *Actívatexto* is the first mobile intervention that promotes smoking cessation and physical activity among Latinos. This work demonstrates that it is feasible to recruit Latino adults who smoke and do not meet the recommended levels of physical activity. The 70% smoking cessation rate seen at month 3 is extremely promising. A previous feasibility study of a mobile intervention that solely promote smoking cessation among Latinos interested in quitting smoking in the next 30 days found a cessation rate of 30% at month 3 (15). A two-arm randomized clinical trial comparing *Actívatexto* to a mobile intervention that solely promotes smoking cessation is warranted to assess the marginal effect of adding physical activity into smoking cessation treatment among Latinos.

The *Actívatexto* intervention increased Fitbit-assessed mean minutes of MVPA per week, although the difference was not statistically significant. Despite nonsignificant statistical results, an average increase of 64 minutes of MVPA per week provides important health benefits (52, 53). This suggests that a mobile intervention that promotes smoking cessation and physical activity may have a positive impact on MVPA and health among Latinos. This study adds valued insights to the expanding literature on promoting physical activity via mobile interventions. Aguiñaga and colleagues, conducted a single-arm pilot study with 20 middle-aged and older Latinos participating in BAILA TECH, a Latin dance program that encompassed Fitbit devices and text messages. The BAILA TECH intervention significantly increased Fitbit-assessed MVPA (*d* = 0.69; ref. 54).

On average, participants adhered to wearing their Fitbit Versa 4 device 69.2% of the days during the 3-month period. This result is appropriate as it falls within the range of previously reported adherence to Fitbit wear. Halliday and colleagues, reported that among parents of children with cancer (*n* = 20) enrolled in a walking intervention, adherence to Fitbit wear at week 12 was 55.2% (49). Moreover, Schumacher and colleagues, reported that among women (*n* = 20) enrolled in a partner-based physical activity program, adherence to Fitbit wear at week 6 was 97% (48).

In this study, participants showed high interest in using NRT, with 88.9% (16/18) of participants using NRT for at least one day and, among those who used NRT for at least one day, used them for 39.6 days. This high level of interest is consistent with new smoking cessation studies among Latinos (16). Moreover, this level of interest challenges older studies that have suggested limited interest in pharmacotherapy use among Latinos (8, 9).

This study provides preliminary evidence that *Actívatexto* has the potential to increase both self-efficacy for smoking cessation and physical activity. This result is relevant given that self-efficacy is one of the most consistent predictors of smoking cessation and physical activity in the literature (55, 56) and an important component of the social cognitive theory (57). Finally, *Actívatexto* was well received by participants, most of whom reported high levels of satisfaction.

Engaging in physical activity can put individuals at risk of injury (58, 59). This study used the PAR-Q to identify individuals who were at risk of injury, making them ineligible to participate in the study. Eight of the 21 individuals who were ineligible to participate in the study (38.1%) were due to the use of the PAR-Q. A pragmatic approach would have been appropriate for individuals who were at risk of injury to receive authorization from their primary care physician to engage in physical activity. Future studies could adopt this pragmatic approach to maximize study participation and make the study results more generalizable.

### Limitations and Strengths

This study has some limitations that should be considered when interpreting the findings. First, the study had a small sample size. However, the enrollment of 20 participants was sufficient to assess the feasibility and acceptability of *Actívatexto*. Second, no comparison group was available, limiting our ability to assess the efficacy of the intervention. Third, biomarkers (e.g., cotinine, carbon monoxide) were not used to verify smoking abstinence, making it possible that the actual cessation rate was lower. However, self-reported smoking abstinence has been judged to be appropriate for minimal contact community interventions that do not involve face-to-face contact (60). Fourth, the study solely focused on MVPA. Future studies should consider promoting and monitoring light intensity physical activity as evidence shows that it provides health benefits (61–63). Fifth, findings should be generalized cautiously. For example, participants were interested in quitting smoking in the next 30 days and study participation was limited to one member per household. Finally, most participants in this study were born in Cuba, the United States, and Puerto Rico, reflecting the Latino groups with the highest smoking rates (4). It remains unknown whether these preliminary results can be generalized to the entire Latino population.

Despite these limitations, this study has several strengths. First, this study builds upon an established history of tobacco cessation research with the Latino community (13–18). Second, this work is grounded in principles of community-based participatory research (64). Community-based participatory research is a partnership approach that involves community members across all phases of research (64). This approach ensures that the research is appropriate, relevant, and meaningful to the Latino community. Third, the study has an appropriate representation of men (45% of the total sample size). This is relevant given the historic underrepresentation of men in studies promoting both smoking cessation and physical activity (24–28). Fourth, the intervention promoted physical activity without relying on highly structured, supervised exercise at research-based fitness facilities, making it more accessible. Finally, the inclusion of Spanish-speaking participants is another study strength. Twelve participants (60%) completed the study in Spanish.

## Conclusion

*Actívatexto*, a mobile intervention that promotes smoking cessation and physical activity among Latinos, resulted in a noteworthy cessation rate at month 3 (70%), increased mean minutes of MVPA per week, produced high use of NRT, increased self-efficacy for smoking cessation and physical activity, and was well received by participants. Additional testing in a formal randomized clinical trial is warranted to assess the efficacy of the intervention.

## References

[1] U.S. Census Bureau 2010 race and ethnicity. Available from: https://www.census.gov/quickfacts/fact/table/US/PST045219.

[2] Projections of the size and composition of the U.S. population: 2014 to 2060. Available from: www.census.gov/content/dam/Census/library/publications/2015/demo/p25–1143.pdf.

[3] Cornelius ME , Loretan CG, Wang TW, Jamal A, Homa DM. Tobacco product use among adults – United States, 2020. MMWR Morb Mortal Wkly Rep 2022;71:397–405. 35298455 10.15585/mmwr.mm7111a1 PMC8942309

[4] Kaplan RC , Bangdiwala SI, Barnhart JM, Castañeda SF, Gellman MD, Lee DJ, . Smoking among U.S. Hispanic/Latino adults: the Hispanic community health study/study of Latinos. Am J Prev Med 2014;46:496–506. 24745640 10.1016/j.amepre.2014.01.014 PMC5794209

[5] Babb S , Malarcher A, Asman K, Johns M, Caraballo R, VanFrank B, . Disparities in cessation behaviors between Hispanic and non-Hispanic White adult cigarette smokers in the United States, 2000–2015. Prev Chronic Dis 2020;17:E10. 31999539 10.5888/pcd17.190279 PMC6993776

[6] Hooper MW , Payne M, Parkinson KA. Tobacco cessation pharmacotherapy use among racial/ethnic minorities in the United States: considerations for primary care. Fam Med Commun Health 2017;5:193–203.

[7] Soto Mas FG , Papenfuss RL, Jacobson HE, Hsu CE, Urrutia-Rojas X, Kane W. Hispanic physicians’ tobacco intervention practices: a cross-sectional survey study. BMC Public Health 2005;5:120. 16287500 10.1186/1471-2458-5-120 PMC1308823

[8] Levinson AH , Borrayo EA, Espinoza P, Flores ET, Pérez-Stable EJ. An exploration of Latino smokers and the use of pharmaceutical aids. Am J Prev Med 2006;31:167–71. 16829334 10.1016/j.amepre.2006.03.022

[9] Levinson AH , Pérez-Stable EJ, Espinoza P, Flores ET, Byers TE. Latinos report less use of pharmaceutical aids when trying to quit smoking. Am J Prev Med 2004;26:105–11. 14751320 10.1016/j.amepre.2003.10.012

[10] Wetter DW , Mazas C, Daza P, Nguyen L, Fouladi RT, Li Y, . Reaching and treating Spanish-speaking smokers through the National Cancer Institute's Cancer Information Service. A randomized controlled trial. Cancer 2007;109:406–13. 17149758 10.1002/cncr.22360

[11] Cox LS , Cupertino AP, Tercyak KP. Interest in participating in smoking cessation treatment among Latino primary care patients. J Clin Psychol Med Settings 2011;18:392–9. 21984387 10.1007/s10880-011-9259-y PMC3229918

[12] Cupertino AP , Cox LS, Garrett S, Suarez N, Sandt H, Mendoza I, . Tobacco use and interest in smoking cessation among Latinos attending community health fairs. J Immigr Minor Health 2011;13:719–24. 20936430 10.1007/s10903-010-9404-y

[13] Arana-Chicas E , Cartujano-Barrera F, Ogedegbe C, Ellerbeck EF, Cox LS, Graves KD, . Feasibility and effectiveness of recruiting latinos in Decídetexto-a smoking cessation clinical trial from an emergency department patient registry. Int J Environ Res Public Health 2021;18:10859. 34682601 10.3390/ijerph182010859 PMC8535914

[14] Arana-Chicas E , Cartujano-Barrera F, Rieth KK, Richter KK, Ellerbeck EF, Cox LS, . Effectiveness of recruitment strategies of Latino smokers: secondary analysis of a mobile health smoking cessation randomized clinical trial. J Med Internet Res 2022;24:e34863. 35759320 10.2196/34863 PMC9274407

[15] Cartujano-Barrera F , Arana-Chicas E, Ramírez-Mantilla M, Perales J, Cox LS, Ellerbeck EF, . “Every day I think about your messages": assessing text messaging engagement among Latino smokers in a mobile cessation program. Patient Prefer Adherence 2019;13:1213–9. 31413549 10.2147/PPA.S209547 PMC6659777

[16] Cartujano-Barrera F , Cox LS, Arana-Chicas E, Ramírez M, Perales-Puchalt J, Valera P, . Feasibility and acceptability of a culturally- and linguistically-adapted smoking cessation text messaging intervention for Latino smokers. Front Public Health 2020;8:269. 32714891 10.3389/fpubh.2020.00269 PMC7344180

[17] Cartujano-Barrera F , Arana-Chicas E, Catley D, Cox LS, Diaz FJ, Ellerbeck EF, . Decídetexto: mobile cessation support for Latino smokers. Study protocol for a randomized clinical trial. Contemp Clin Trials 2020;99:106188. 33080379 10.1016/j.cct.2020.106188 PMC8315307

[18] Cartujano-Barrera F , Peña-Vargas CI, Arana-Chicas E, Pérez-Ramos JG, Mattei J, Hurtado-de-Mendoza A, . Decídetexto: feasibility and acceptability of a mobile smoking cessation intervention in Puerto Rico. Int J Environ Res Public Health 2021;18:1379. 33546156 10.3390/ijerph18041379 PMC7913140

[19] Haasova M , Warren FC, Ussher M, Van Rensburg KJ, Faulkner G, Cropley M, . The acute effects of physical activity on cigarette cravings: systematic review and meta-analysis with individual participant data. Addiction 2013;108:26–37. 22861822 10.1111/j.1360-0443.2012.04034.x

[20] Roberts V , Gant N, Sollers JJ 3rd, Bullen C, Jiang Y, Maddison R. Effects of exercise on the desire to smoke and physiological responses to temporary smoking abstinence: a crossover trial. Psychopharmacol 2015;232:1071–81. 10.1007/s00213-014-3742-8 25266608

[21] Roberts V , Maddison R, Simpson C, Bullen C, Prapavessis H. The acute effects of exercise on cigarette cravings, withdrawal symptoms, affect, and smoking behaviour: systematic review update and meta-analysis. Psychopharmacol 2012;222:1–15. 10.1007/s00213-012-2731-z 22585034

[22] Ussher M , Nunziata P, Cropley M, West R. Effect of a short bout of exercise on tobacco withdrawal symptoms and desire to smoke. Psychopharmacol 2001;158:66–72. 10.1007/s002130100846 11685385

[23] Audrain-McGovern J , Strasser AA, Ashare R, Wileyto EP. Reinforcing value of smoking relative to physical activity and the effects of physical activity on smoking abstinence symptoms among young adults. Psychopharmacol 2015;23:477–85. 10.1037/pha0000051 PMC4658299 26348158

[24] Ussher MH , Faulkner GEJ, Angus K, Hartmann-Boyce J, Taylor AH. Exercise interventions for smoking cessation. Cochrane Database Syst Rev 2019;2019:CD002295. 31684691 10.1002/14651858.CD002295.pub6 PMC6819982

[25] Marcus BH , Albrecht AE, King TK, Parisi AF, Pinto BM, Roberts M, . The efficacy of exercise as an aid for smoking cessation in women: a randomized controlled trial. Arch Intern Med 1999;159:1229–34. 10371231 10.1001/archinte.159.11.1229

[26] Marcus BH , Albrecht AE, Niaura RS, Abrams DB, Thompson PD. Usefulness of physical exercise for maintaining smoking cessation in women. Am J Cardiol 1991;68:406–7. 1858687 10.1016/0002-9149(91)90843-a

[27] Bock BC , Fava JL, Gaskins R, Morrow KM, Williams DM, Jennings E, . Yoga as a complementary treatment for smoking cessation in women. J Womens Health 2012;21:240–8. 10.1089/jwh.2011.2963 PMC3304243 21992583

[28] Martin JE , Calfas KJ, Patten CA, Polarek M, Hofstetter CR, Noto J, . Prospective evaluation of three smoking interventions in 205 recovering alcoholics: one- year results of Project SCRAP-Tobacco. J Consult Clin Psychol 1997;65:190–4. 9103749 10.1037//0022-006x.65.1.190

[29] Cleland C , Ferguson S, Ellis G, Hunter RF. Validity of the international physical activity questionnaire (IPAQ) for assessing moderate-to-vigorous physical activity and sedentary behaviour of older adults in the United Kingdom. BMC Med Res Methodol 2018;18:176. 30577770 10.1186/s12874-018-0642-3 PMC6303992

[30] Medina C , Barquera S, Janssen I. Validity and reliability of the international physical activity questionnaire among adults in Mexico. Rev Panam Salud Publica 2013;34:21–8. 24006016

[31] Adams R . Revised physical activity readiness questionnaire. Can Fam Physician 1999;995:1004–5. PMC2328306 10216799

[32] Schwartz J , Mas-Alòs S, Takito MY, Martinez J, Álvarez Cueto ME, Rubio Mibelli MS, . Cross-cultural translation, adaptation, and reliability of the Spanish version of the physical activity readiness questionnaire for everyone (PAR-Q+). Health Fitness J Canada 2019;12:3–14.

[33] Hernández-Torres R , Alaniz-Cantu E, Bautista Rojas MV, Lara D, Merritt S, DeJesus E, . Understanding the perspectives of Latino adults who smoke on physical activity: a qualitative study. Int J Environ Res Public Health 2023;20:3128. 36833833 10.3390/ijerph20043128 PMC9964119

[34] Musa SB , Ellis R, Chafe B, Sturrock SL, Maher RA, Cullen K, . Wearable device validity in measuring steps, energy expenditure, and heart rate across age, gender, and body mass index: data analysis from a systematic review. J Phys Act Health 2022;20:100–5. 36535270 10.1123/jpah.2022-0160

[35] Sasaki JE , Hickey A, Mavilia M, Tedesco J, Dinesh J, Keadle SK, . Validation of the Fitbit wireless activity tracker for prediction of energy expenditure. J Phys Act Health 2015;12:149–54. 24770438 10.1123/jpah.2012-0495

[36] Takacs J , Pollock CL, Guenther JR, Bahar M, Napier C, Hunt MA. Validation of the Fitbit one activity monitor device during treadmill walking. J Sci Med Sport 2014;17:496–500. 24268570 10.1016/j.jsams.2013.10.241

[37] Case MA , Burwick HA, Volpp KG, Patel MS. Accuracy of smartphone applications and wearable devices for tracking physical activity data. JAMA 2015;313:625–6. 25668268 10.1001/jama.2014.17841

[38] Tobacco Use and Dependence Guideline Panel. Treating tobacco use and dependence: 2008 update. Rockville (MD): US Department of Health and Human Services; 2008.

[39] Etter J , Bergman M, Humair J, Perneger T. Development and validation of a scale measuring self-efficacy of current and former smokers. Addiction 2000;95:901–13. 10946439 10.1046/j.1360-0443.2000.9569017.x

[40] Cartujano-Barrera F , McIntosh S, Cox LS, Arana-Chicas E, Catley D, Ellerbeck EF, . Translation and examination of the reliability and validity of the Spanish version of the smoking self-efficacy questionnaire among Latino smokers. Tob Use Insights 2021;14:1179173X211035366. 10.1177/1179173X211035366 PMC8326995 34377041

[41] Pekmezi D , Jennings E, Marcus BH. Evaluating and enhancing self-efficacy for physical activity. ACSMs Health Fit J 2009;13:16–21. 29910597 10.1249/FIT.0b013e3181996571 PMC6003667

[42] Mendoza-Vasconez AS , Marquez B, Benitez TJ, Marcus BH. Psychometrics of the self-efficacy for physical activity scale among a Latina women sample. BMC Public Health 2018;18:1097. 30185171 10.1186/s12889-018-5998-0 PMC6125999

[43] Riebe D , Ehrman JK, Liguori G, Magal M. Chapter 6 general principles of exercise prescription. ACSM's guidelines for exercise testing and prescription. 10th ed. Philadelphia (PA): Wolters Kluwer/Lippincott Williams & Wilkins; 2018. p. 143–79.

[44] World Health Organization. Physical activity. Available from: www.who.int/news-room/fact-sheets/detail/physical-activity.

[45] Haskell WL , Lee IM, Pate RR, Powell KE, Blair SN, Franklin BA, . Physical activity and public health: updated recommendation for adults from the American college of sports medicine and the American Heart Association. Med Sci Sports Exerc 2007;39:1423–34. 17762377 10.1249/mss.0b013e3180616b27

[46] Centers for Disease Control and Prevention. How much physical activity do adults need?. Available from: www.cdc.gov/physicalactivity/basics/adults/index.htm.

[47] St Fleur RG , St George SM, Leite R, Kobayashi M, Agosto Y, Jake-Schoffman DE. Use of Fitbit devices in physical activity intervention studies across the life course: narrative review. JMIR Mhealth Uhealth 2021;9:e23411. 34047705 10.2196/23411 PMC8196365

[48] Schumacher LM , Arigo D, Thomas C. Understanding physical activity lapses among women: responses to lapses and the potential buffering effect of social support. J Behav Med 2017;40:740–9. 28382571 10.1007/s10865-017-9846-y

[49] Halliday GC , Miles GCP, Marsh JA, Kotecha RS, Alessandri AJ. Regular exercise improves the well-being of parents of children with cancer. Pediatr Blood Cancer 2017;64. 10.1002/pbc.26668 28627013

[50] Schrager JD , Shayne P, Wolf S, Das S, Patzer RE, White M, . Assessing the influence of a Fitbit physical activity monitor on the exercise practices of emergency medicine residents: a pilot study. JMIR Mhealth Uhealth 2017;5:e2. 28143805 10.2196/mhealth.6239 PMC5309436

[51] West R , Hajek P, Stead L, Stapleton J. Outcome criteria in smoking cessation trials: proposal for a common standard. Addiction 2005;100:299–303. 15733243 10.1111/j.1360-0443.2004.00995.x

[52] Warburton DER , Bredin SSD. Health benefits of physical activity: a systematic review of current systematic reviews. Curr Opin Cardiol 2017;32:541–56. 28708630 10.1097/HCO.0000000000000437

[53] Anderson E , Durstine JL. Physical activity, exercise, and chronic diseases: a brief review. Sports Med Health Sci 2019;1:3–10. 35782456 10.1016/j.smhs.2019.08.006 PMC9219321

[54] Aguiñaga S , Marques IG, Kitsiou S, Balbim GM, Gerber BS, Buchholz SW, . BAILAMOS with mhealth technology! improving physical activity and well-being in middle-aged and older latinxs: a pre-post feasibility study. Health Educ Behav 2021;48:575–83. 34521228 10.1177/10901981211027517

[55] Gwaltney CJ , Metrik J, Kahler CW, Shiffman S. Self-efficacy and smoking cessation: a meta-analysis. Psychol Addict Behav 2009;23:56–66. 19290690 10.1037/a0013529 PMC3829471

[56] Neupert SD , Lachman ME, Whitbourne SB. Exercise self-efficacy and control beliefs: effects on exercise behavior after an exercise intervention for older adults. J Aging Phys Act 2009;17:1–16. 19299835 10.1123/japa.17.1.1 PMC3740728

[57] Bandura A . Social cognitive theory: an agentic perspective. Annu Rev Psychol 2001;52:1–26. 11148297 10.1146/annurev.psych.52.1.1

[58] Jones BH , Cowan DN, Knapik JJ. Exercise, training and injuries. Sports Med 1994;18:202–14. 7809556 10.2165/00007256-199418030-00005

[59] Hauret KG , Bedno S, Loringer K, Kao TC, Mallon T, Jones BH. Epidemiology of exercise- and sports-related injuries in a population of young, physically active adults: a survey of military servicemembers. Am J Sports Med 2015;43:2645–53. 26378031 10.1177/0363546515601990

[60] Benowitz NL , Bernert JT, Foulds J, Hecht SS, Jacob P, Jarvis MJ, . Biochemical verification of tobacco use and abstinence: 2019 update. Nicotine Tob Res 2020;22:1086–97. 31570931 10.1093/ntr/ntz132 PMC7882145

[61] Buman MP , Hekler EB, Haskell WL, Pruitt L, Conway TL, Cain KL, . Objective light-intensity physical activity associations with rated health in older adults. Am J Epidemiol 2010;172:1155–65. 20843864 10.1093/aje/kwq249 PMC3004766

[62] Loprinzi PD . Objectively measured light and moderate-to-vigorous physical activity is associated with lower depression levels among older US adults. Aging Ment Health 2013;17:801–5. 23731057 10.1080/13607863.2013.801066

[63] Loprinzi PD , Ramulu PY. Objectively measured physical activity and inflammatory markers among US adults with diabetes: implications for attenuating disease progression. Mayo Clin Proc 2013;88:942–51. 24001486 10.1016/j.mayocp.2013.05.015

[64] Israel BA , Schulz AJ, Parker EA, Becker AB. Review of community-based research: assessing partnership approaches to improve public health. Annu Rev Public Health 1998;19:173–202. 9611617 10.1146/annurev.publhealth.19.1.173

